# Epigenetics in Schistosomes: What We Know and What We Need Know

**DOI:** 10.3389/fcimb.2016.00149

**Published:** 2016-11-11

**Authors:** Weiwei Liu

**Affiliations:** Department of Avian Diseases, Shanghai Veterinary Research Institute, Chinese Academy of Agricultural ScienceShanghai, China

**Keywords:** schistosome, DNA methylation, histone modifying enzymes, microRNAs, drug discovery

## Abstract

Schistosomes are metazoan parasites and can cause schistosomiasis. Epigenetic modifications include DNA methylation, histone modifications and non-coding RNAs. Some enzymes involved in epigenetic modification and microRNA processes have been developed as drugs to treat the disease. Compared with humans and vertebrates, an in-depth understanding of epigenetic modifications in schistosomes is starting to be realized. DNA methylation, histone modifications and non-coding RNAs play important roles in the development and reproduction of schistosomes and in interactions between the host and schistosomes. Therefore, exploring and investigating the epigenetic modifications in schistosomes will facilitate drug development and therapy for schistosomiasis. Here, we review the role of epigenetic modifications in the development, growth and reproduction of schistosomes, and the interactions between the host and schistosome. We further discuss potential epigenetic targets for drug discovery for the treatment of schistosomiasis.

## Introduction

Schistosomiasis is a parasitic disease that is caused by schistosomes. More than 200 million people are infected with schistosomes worldwide. However, only one drug, praziquantel, is available for the treatment of this condition, and drug resistance to praziquantel is an important concern (Bergquist et al., 2008). Therefore, the identification of new drug targets is urgently needed. Schistosomes are digenean parasites, which can infect intermediate hosts (snails) and definitive hosts (vertebrates). Schistosomes can live in the body of the definitive host for several decades, suggesting that expression and regulation of genes could affect their development and reproduction. Epigenetics is an important feature of gene regulation and expression. Some enzymes that are involved in epigenetic modifications have been developed as drug targets against human disease, including two histone deacetylase (HDAC) inhibitors (Arrowsmith et al., 2012). Therefore, a detailed understanding of the epigenetics of schistosomes and an exploration of the possible underlying mechanisms will provide useful information for understanding the epigenetics of schistosomes and the development of drugs against schistosomiasis.

**Figure 1:**
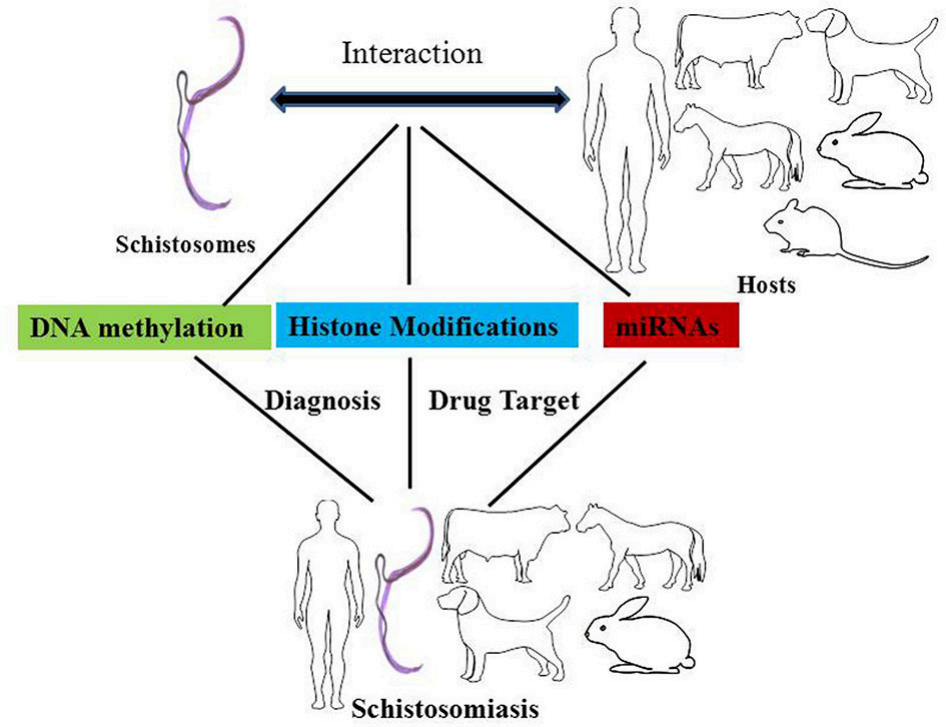
**The effects of epigenetic modifications on the interaction between schistosomes and hosts, drug targets against schistosomiasis, and biomarkers for the diagnosis of schistosomiasis**. DNA methylation, histone modifications, and miRNAs play important roles in epigenetic modifications in the interaction between schistosomes and hosts. DNA methyltransferases, histone acetyltransferases (HATs), and histone deacetylase are potential drug targets against schistosomiasis. In addition, the levels of DNA methylation and miRNAs can be used as diagnostic biomarkers for schistosomiasis.

In a broad sense, epigenetics generally refers to changes in gene expression that are stable between cell divisions, and sometimes between generations, but these changes do not involve alterations in the underlying DNA sequence of the organism (Jiang et al., 2004; Portela and Esteller, 2010). The carriers of epigenetic information are DNA methylation, histone modifications, and non-coding RNAs, the relocation of a nucleus to a nuclear compartment and modifications of this information might affect different processes, including genome stability. Here, we will discuss the mechanisms underlying DNA methylation and the modification of histones and microRNAs, and their potential roles in schistosomes and in therapies designed to treat schistosomiasis (Figure 1).

## Mechanisms

### DNA methylation

**Figure 2:**
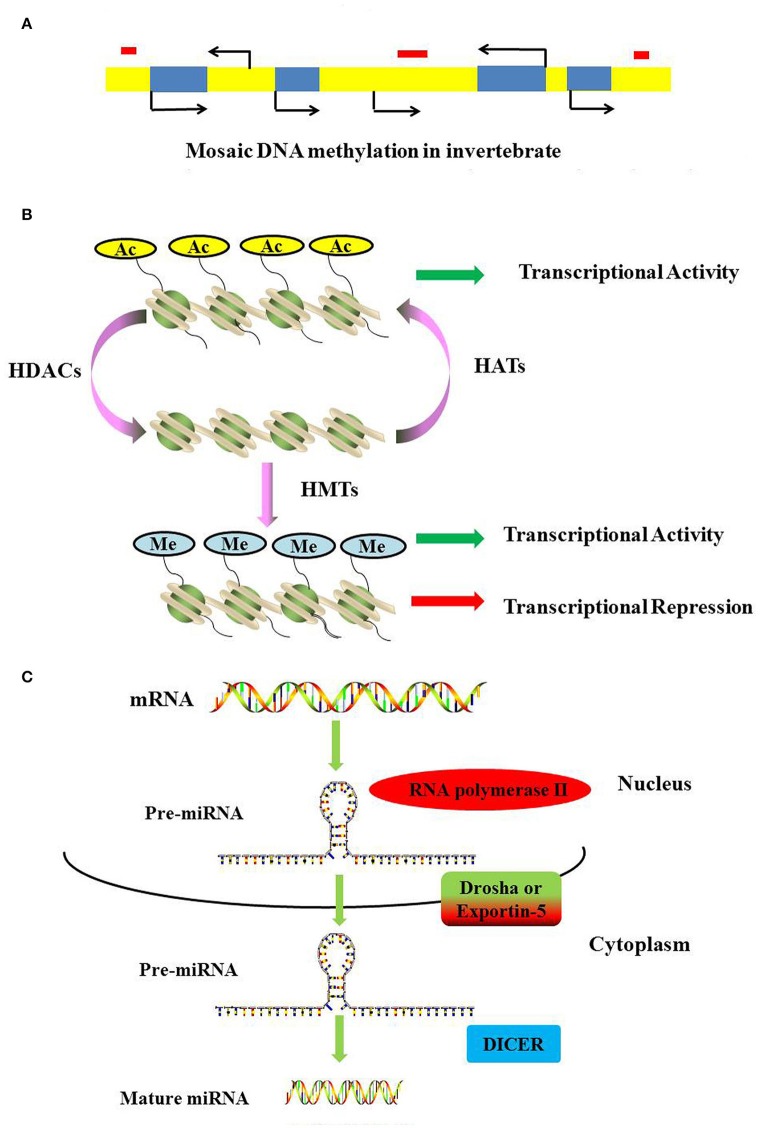
**Mechanisms of DNA methylation, histone modifications and miRNAs. (A)** DNA methylation landscapes in invertebrates. Mosaic methylation is characteristic of most invertebrates. Mosaic methylation comprises domains of heavily methylated DNA interspersed with domains that are free of methylation. Mosaic DNA methylation consists of stable methylated (yellow) and unmethylated (blue) domains. The stable methylated and unmethylated domains are interspersed. Transposable elements are frequently unmethylated and match the methylation status of the surrounding DNA (red box). **(B)** Methylation and acetylation of histones. Histone acetylation can neutralize the positive charge of lysine, leading to a more relaxed structure of chromatin. The transcriptional machinery recruitment is permitted, and consequently transcription is activated. Histone methylation has been shown to be present during both transcriptional activation and repression. **(C)** The generation of miRNAs. The microRNAs are generated from long, capped, hairpin and polyadenylated pre-miRNAs, which are usually transcribed by RNA polymerase II (Pol II). Pre-miRNAs are processed by Drosha or Exportin-5, and then they are transported into the cytoplasm to be processed by RNase Dicer into mature double-stranded miRNAs.

DNA methylation requires the participation of DNA methyltransferases. In this process, three DNA methyltransferases (Dnmts) establish (Dnmt3a and Dnmt3b) and maintain (Dnmt1) DNA methylation (Hamidi et al., 2015). DNMT1 is considered to maintain the methyltransferase that is responsible for copying DNA methylation patterns to the daughter strands during DNA replication; DNMT3a and DNMT3b are considered to be the de novo methyltransferases that construct DNA methylation patterns and are essential for mammalian development; Dnmt2 is primarily a tRNA methyltransferase with only weak DNA methyltransferase activity (Liyanage et al., 2014; Meng et al., 2015). In vertebrates, ~70–80% of cytosines in CpG islands are methylated (Jabbari and Bernardi, 2004). As a general rule, methylated cytosines recruit methylated CpG-binding proteins (MBDs) to alter the chromatin structure and silence gene expression (Lo and Weksberg, 2014). In contrast, most invertebrates display a wide range of DNA methylation in the form of a mosaic pattern (Suzuki et al., 2007; Suzuki and Bird, 2008). In invertebrates, DNA methylation may not be limited to the canonical CpG targets but is also present as a mosaic pattern, referring to tracts of methylated CpGs that are interspersed with unmethylated regions across the genome (Figure 2A). Moreover, methylated genes can be actively transcribed via alternative splicing in several invertebrates (Flores et al., 2012).

In addition to the methylation or hydroxymethylation of cytosine residues, DNA methylation in lower eukaryotes also involves the modification of adenine to N6-methyladenine (m6A) (Tchurikov, 2005; Ratel et al., 2006), such as *Trypanosoma brucei* TbPRMT6, which, like human PRMT6, catalyzes the production of monomethylarginine and asymmetric dimethylarginine residues (Lott et al., 2014; Wang et al., 2014).

### Modification of histones

The modification of histones often takes place at the tails of the histone (Imhof, 2006; Lennartsson and Ekwall, 2009). The varieties of possible histone modifications include phosphorylation, ubiquitinylation, sumoylation, acetylation and methylation (Berger, 2002; Peterson and Laniel, 2004).

Histone acetylation is processed by histone acetyltransferases (HATs) and transfers an acetyl residue to lysines, particularly in the N-terminal tails of histones H3 and H4 (Peterson and Laniel, 2004). Acetyl-CoA is used as a co-factor in this process. In contrast, HDAC removes the acetylation of histone. In humans, 18 HDACs have been identified and classified according to their co-factor dependency on either zinc or NAD+ and their sequence homology to yeast proteins (Marks and Xu, 2009). Classes I, II, and IV have structurally related catalytic domains and a Zn^2+^-dependent catalytic mechanism (Gregoretti et al., 2004). The class III HDACs, or sirtuins, are phylogenetically unrelated and rely on NAD+ as a co-factor (Greiss and Gartner, 2009). Histone acetylation can result in a more relaxed structure of chromatin, and the transcriptional machinery is permitted to recruit and consequently activate transcription (Figure 2B).

Histone methylation is completed by S-adenosylmethionine-dependent methyltransferase and occurs on lysine or arginine residues in histone tails (Beaver and Waters, 2016). Histone methylation has been shown to play a role in both transcriptional activation and repression (Wang et al., 2001; Jeong et al., 2011; Kallestad et al., 2014; Figure 2B). Histone demethylation is mediated by two classes of demethylase: the Jumonji family and the flavin-dependent lysine-specific demethylase1 (KDM1/LSD1) and 2 (KDM2/LSD2) (Pedersen and Helin, 2010).

Histone phosphorylation is a dynamic modification in which a phosphate group is added to a specific amino acid residue such as serine, threonine or tyrosine. Histone phosphorylation is implicated in multiple cellular processes. For example, phosphorylation of histone H1, H3, H2AX, and H2B is involved in the regulation of transcription, mitotic chromatin condensation, DNA damage responses and apoptosis, respectively (Hans and Dimitrov, 2001; Celeste et al., 2003; Cheung et al., 2003; Nowak and Corces, 2004; Bungard et al., 2010; Sharma et al., 2012).

### Non-coding RNAs

Non-coding RNAs are classes of functional RNAs that do not encode protein but have important roles in translation, gene regulation, and RNA splicing, among others (Santosh et al., 2015). Non-coding RNA genes include highly abundant and functionally important RNAs, such as tRNA, rRNA, snoRNA, microRNA, siRNA, piRNA, and lncRNA. Among these, microRNAs are the best characterized. A microRNA (miRNA) is a small non-coding RNA molecule consisting of approximately 21–25 nucleotides that functions in a wide range of pivotal biological processes such as development, cell proliferation and differentiation, cell death, metabolism, and signal transduction (Carrington and Ambros, 2003; Hwang and Mendell, 2006; Fendler et al., 2011; Sayed and Abdellatif, 2011). MicroRNAs are generated from long, capped, hairpin and polyadenylated pre-miRNAs, which are usually transcribed by RNA polymerase II (Pol II). Pre-miRNAs are processed by Drosha or Exportin-5 and then transported into the cytoplasm where they are processed by the RNase Dicer into mature, double-stranded miRNAs (Figure 2C). MiRNAs usually regulate transcript levels by binding to the 3′UTR of their target mRNAs, resulting in mRNA degradation or translational inhibition.

MiRNAs play important roles in the regulation of gene expression, but a subgroup of these ncRNAs (defined as epi-miRNAs) directly and indirectly control the expression of epigenetic effectors such as DNMTs and HDACs, shedding new light on the functions of miRNAs as both genetic and epigenetic regulators (Valeri et al., 2009; Fabbri and Calin, 2010; Sato et al., 2011). Based on the term epi-miRNAs, these miRNAs directly or indirectly target effectors of the epigenetic machinery. For example, HDAC4 has been confirmed to be one of the important targets of miR-140 (Song et al., 2009), whereas miR-449a binds to the 3′UTR of HDAC1 (Noonan et al., 2009; Liu et al., 2015).

## Epigenetic studies in schistosomes: what is known?

### DNA methylation

Previously, the genome of *Schistosoma mansoni* was considered to lack detectable DNA methylation patterns (Raddatz et al., 2013). Recently, cytosine methylation was found to be a conserved epigenetic feature throughout the phylum Platyhelminthes, including *Schistosoma haematobium, Schistosoma japonicum*, and *S. mansoni* (Geyer et al., 2013). In addition to cytosine methylation, the arginine methyltransferase PRMT1 homolog was identified in *S. mansoni* and *S. japonicum* (Mansure et al., 2005; Diao et al., 2014). Dnmt2 and methyl-CpG-binding domain proteins (MBD) are present in the *S. mansoni* genome, and methylation deficiencies of the schistosome genome in this epigenetic process affect oviposition and ovarian morphology in adult worm pairs treated with the DNA methyltransferase inhibitor 5-AzaC (Geyer et al., 2011). These findings further demonstrate that DNA methylation machinery exists in different lifecycle stages, that methylation occurs in a highly repetitive intronic region and that targeting cytosine methylation components with selective demethylating agents provides a promising new avenue to combat schistosomiasis.

DNA methylation of the host was modified after schistosome infection. For example, CpG island methylation in schistosoma-associated bladder cancer displays a higher methylation index compared with that in non-schistosoma-associated bladder cancer(Gutierrez et al., 2004); during *S. mansoni* infection, a population of IFN-γ/IL-4 double-positive cells was found to display a discrete DNA methylation pattern in CpG islands within the body of the Gata3 gene, which encodes the master regulator of Th2 identity. Furthermore, expression of the Gata3 gene in IFN-γ (+) IL-4(+) cells was lower than that in IFN-γ (+) IL-4(−) (Th1) and IFN-γ (−) IL-4(+) (Th2) cells (Deaton et al., 2014). The DNA methylation state can be used as a biomarker for schistosome infection. In *S. haematobium*, the promoter of RASSF1A and TIMP3 is hypermethylated and can serve as a biomarker of *S. haematobium* infection (Zhong et al., 2013).

### Modification of histones

To date, only a few studies have been conducted to investigate histone marks and their roles in schistosome development and reproduction. However, histone modifications have been identified in different life stages of schistosomes, and changes in histone modifications appear to be crucial for pathogenesis and thus to represent therapeutic targets. For example, bivalent histone H3 methylation was observed in cercariae, demethylation of H3K27 and activation of transcription were observed during the transformation into schistosomula (remaining absent in adults), and alterations of H3K9 methylation and acetylation occurred upstream and downstream of the transcriptional start site (TSS) (Roquis et al., 2015). In schistosomes, sex is genetically determined by the presence of sex chromosomes: ZZ in males or ZW in females and the phenotypic sexual dimorphism appears only after infection of the vertebrate definitive host. Picard et al. compared H3K27me3 histone modification before (in cercariae) and after (in adults) the phenotypic sexual dimorphism appearance (Picard et al., 2016). The H3K27me3 enrichment profile in cercariae differs between the two sexes both upstream and along the transcription unit, whereas in the adult stage, males and females display the same profile after the TSS, while their profile upstream the TSS remains different (Picard et al., 2016). These results suggest epigenetic regulators play important roles in the sex determination and sexual differentiation in schistosomes and represent a promising source of therapeutic targets. In the adaption between the schistosome and its intermediate host, histone modifications also play important roles. The modification profiles of four histone modifications (H3K4me3, H3K27me3, H3K27ac, and H4K20me1) were different between the sympatric host and allopatric host and these histone marks also differ in cercariae and adult (Roquis et al., 2016). The promoters of the *S. mansoni* mucin gene (SmPoMuc), a key component of the compatibility between the schistosome and its snail host, contains the epigenetic marks H3K9Met3 and H3K9Ac, which differ significantly between compatible (C) and incompatible (IC) strains and negatively regulates the expression of SmPoMuc (Fneich et al., 2016). The structure of the parasite chromatin differentially modifies the transcription of SmPoMuc in the IC strain compared with the C strain (Perrin et al., 2013). These findings suggest that histone alterations may be important for the initial steps in the adaptation of pathogens to new hosts and epidrugs can be used to control parasite development. The identification of HDACs and HAT in schistosomes has also revealed the importance of HDACs and HAT as potential therapeutic targets. Based on evidence for the expression of all *S. mansoni* histones among the entire set of public ESTs, five histone families (H1, H2A, H2B, H3, and H4) were detected during all six life cycle stages of the parasite (with the exception of H4 in germ balls, the second least sequenced stage) (Anderson et al., 2012). The HATs, GCN5, was identified in *S. mansoni*, and specific acetylation at H3K14 was catalyzed by the transcriptional co-activator GCN5 (de Moraes Maciel et al., 2004, 2008). Sm CBP/p300 was also identified in *S. mansoni*, and it was expressed during all life cycle stages, interacted functionally with the nuclear receptor SmFtz-F1 and also potentiated the transcriptional activity of this receptor in the CV-1 cell line (Bertin et al., 2006). Knocking down SmGCN5 or SmCBP1 significantly decreased the transcription and protein synthesis of Smp14, an eggshell protein in the mature female worm, damaging the reproductive system of mature female worms, egg-laying and egg morphology (Carneiro et al., 2014). This result suggests that inhibition of Smp14 expression targeting SmGCN5 and/or SmCBP1 represents a novel and effective strategy to control *S. mansoni* egg development and that HATs can be used as a drug target against schistosomiasis. Regarding HDAC, the NAD^+^-dependent HDAC, smSirt2 in *S. mansoni* was identified using fluorescence-based screening assays (Schiedel et al., 2015). The class I HDAC s, SmHDAC1, 3, and 8, were identified in *S. mansoni* (Oger et al., 2008; Marek et al., 2013; Singh and Pandey, 2015). Additionally, inhibitors of HDACs (HDACi) like TSA and valproic acid can induce schistosome death (adult worms or schistosomula larvae) in culture and increase caspase 3/7 activity (Dubois et al., 2009). This finding provides preliminary information regarding the potential of schistosome HDAC as a potential drug target for schistosomiasis.

### Schistosome miRNAs

Because miRNAs act as critical post-transcriptional regulators in many organisms, studies have been conducted to determine the roles of miRNAs in schistosomes. These miRNAs are likely to play critical roles in schistosome development and gene regulation. To date, a total of 79 mature miRNAs in *S. japonicum* and 225 mature miRNAs in *S. mansoni* have been characterized in miRBase. Five schistosome-specific miRNAs, including four known miRNAs (Bantam, miR-3479, miR-10 and miR-3096) and one novel miRNA (miR-0001, miRBase ID: sja-miR-8185), have been characterized as schistosome-specific small RNA populations by Cheng et al. (Cheng et al., 2013). Different miRNAs participate in the different developmental stages of schistosomes. For example, in *S. japonicum*, sja-miR-71b-5p, sja-miR-1, sja-miR-36-3p, and sja-miR-124-3p were the most abundant miRNAs during the egg stage (Cai et al., 2013); sja-bantam expression was highest in the cercaria stage (Xue et al., 2008); and sja-miR-71 was highest during both the cercaria stage and the egg stage (Xue et al., 2008; Cai et al., 2011). In *S. mansoni*, 112 miRNAs (including 84 novel miRNA families) have been reported in adult worms of *S. mansoni* (Marco et al., 2013); miR-4, miR-6, miR-9, miR-32, miR-125, miR-3, and miR-5 are expressed in adult worms only, and miR-20, miR-18, miR-22, miR-26 and bantam are expressed in schistosomula only (Simões et al., 2011). Thirteen microRNAs exhibit sex-biased expression, 10 of which are more abundant in females than in males, such as mir-31, mir-8447, bantam and mir-8437 (Marco et al., 2013). The miRNAs bantam and miR-31 are associated with the morphological formation of ovaries in female schistosomes in *S. japonicum* (Zhu et al., 2016). These results demonstrate that during the life cycle of schistosomes, different microRNAs are likely to participate in differentiation/maintenance processes. The miRNAs have been shown to be different in distinct hosts. Compared with permissive BALB/c mice, rats are less susceptible to *S. japonicum* infection and are considered to provide an unsuitable microenvironment for parasite growth and development. Forty-one differentially expressed miRNAs have been identified in comparisons of rats and mice (Han et al., 2015). One hundred sixty-two miRNAs are expressed in *M. fortis* in mice, with 12 in the liver, 32 in the spleen and 34 in the lung being differentially expressed in *M. fortis* (Han et al., 2013). These results indicate that some differentially expressed miRNAs may impact the survival and development of the parasite within different hosts. MiRNAs also play roles in the host after schistosome infection. The altered levels of miR-706 and miR-134-5p between uninfected and infected mice are associated with altered expression levels of the Caspase-3 and Creb1 genes, respectively (Zhu et al., 2015). MiR-454, a target miRNA of Smad4, is down-regulated in the *S. japonicum*-induced murine liver in models of fibrosis, while the expression of Smad4 is up-regulated. These results suggest that circulating miRNAs may serve as important mediators of the pathology of hepatic schistosomiasis. Furthermore, the miRNA profiles in different tissues (liver, spleen, and lung) of *S. japonicum*-infected mice suggest that miRNAs may be involved in the regulation of several signaling pathways—such as the MAPK, insulin, Toll-like receptor, and TGF-β pathways—during schistosome infection (Han et al., 2013). Inhibition of microRNA-21 can protect mice against lethal schistosome infection by repressing both the IL-13 and transforming growth factor beta 1 (TGFβ1) pathways (Sombetzki et al., 2015). These results suggest that some signaling pathways in the host may be affected by schistosome infection. Circulating miRNAs, which are present in a stable form in the plasma or serum, have been considered to be ideal biomarkers for the diagnosis of some cancers. It is possible that such circulating miRNAs could also serve as biomarkers for the diagnosis of schistosomiasis. Three of the parasite-derived miRNAs (miR-277, miR-3479-3p and bantam) in host serum can be used as novel biomarkers of *S. mansoni* infection (Hoy et al., 2014). The expression levels of serum miR-223 are significantly elevated after infection, but they return to nearly normal levels following treatment with praziquantel (PZQ) (He et al., 2013), implying that murine miR-223 may be a biomarker for schistosome infection.

## Epigenetic regulation in schistosomes: what knowledge is needed?

The availability of the genome sequence of schistosomes has been a useful tool for the investigation of epigenetic modification in schistosomes. The epigenomic profile of schistosomes remains poorly understood, although great advances have been achieved in the epigenome of model organisms such as the human and mammalian brain (Lister et al., 2013; Wen et al., 2014). Currently, great advancements have been achieved in EPIGENOME and ENCODE in humans to illustrate the epigenetics and DNA elements in the human genome (Birney et al., 2007; Gerstein et al., 2012; Schultz et al., 2015; Tang et al., 2015). In comparison to the advancements in humans, the DNA elements and transcription factors that are involved in gene expression and regulation in schistosomes remain to be elucidated. The schistosome life cycle is complex; however, the epigenetic profiles of these developmental stages are not clear and may differ in different hosts. Therefore, an exploration of the epigenetic profile during different development stages and in different hosts is needed. Epigenetic modifications can regulate gene expression, yet the mechanism(s) by which epigenetic modifications in schistosomes regulates gene expression in the host or the parasite remains unclear. In addition, although the enzymes involved in DNA methylation have been identified in schistosomes, the mechanisms responsible for DNA demethylation in schistosomes have not been determined. MiRNAs are best characterized in terms of their functions and implications, but recently a new class of non-coding RNA lncRNAs was identified in the genome. Long non-coding RNAs (long ncRNAs, lncRNAs) are non-protein-coding transcripts that are longer than 200 nucleotides (Mercer et al., 2009). The lncRNAs also have roles in epigenetic regulation (Vadaie and Morris, 2013; Chen and Xue, 2016). For example, one ncRNA, termed HOTAIR, which is a HOX antisense intergenic RNA, interacts with key epigenetic regulators such as histone methyltransferase PRC2 and histone demethylase LSD1 and regulates gene silencing (Tsai et al., 2010). Although lncRNA has been described in other organisms, its role in schistosomes remains unknown. In addition, although some microRNAs have been identified in schistosomes, epi-miRNAs that control the expression of epigenetic effectors, such as DNMTs and HDACs, have not been recognized in schistosomes.

HAT and HDAC have been recognized in schistosomes; however, the molecules involved in histone methylation, histone phosphorylation and other histone modifications in schistosomiasis remain undescribed. Furthermore, the histone marks and their possible roles in schistosome development and reproduction require further elucidation in future studies.

Snails serve as the obligate intermediate host of this parasite and might be manipulated to block the development of intramolluscan larval stages of the parasite. Therefore, the epigenome of the snail has impact on the schistosome infection. Although 5-methyl-cytosine and 5-hydroxy-methyl-cytosine were found in the genome of *Biomphalaria glabrata* (a snail intermediate host for *S. mansoni*) and HSP 70 in the snail was considered as a novel intervention target since epigenetic modifications at the HSP 70 locus influence B. glabrata susceptibility to *S. mansoni*, the knowledge on the snail epigenetics needs to be further investigated (Fneich et al., 2013; Knight et al., 2016).

## Drug targets of epigenetic regulation to control schistosomiasis

Only one drug, praziquantel, is currently available for the treatment and control of schistosomiasis, and the increasing risk of schistosomes that are resistant to praziquantel indicates that the development of new drugs is urgent (Wang et al., 2012). Numerous histone modifying enzyme (HME) inhibitors are under investigation as potential anticancer agents. The HMEs represent particularly promising targets for the development of alternatives to praziquantel. The crystal structure of inhibited histone deacetylase 8 (HDAC8) has been elucidated, and a newly identified smHDAC8 inhibitor has the capacity to induce apoptosis and mortality in schistosomes (Marek et al., 2013). These findings define the framework for the design of small molecule inhibitors that specifically interfere with schistosome epigenetic mechanisms. Sirtuins are NAD+-dependent lysine deacetylases that are involved in a wide variety of cellular processes including histone deacetylation, and they have been shown to be therapeutic targets in various pathologies including cancer. All *S. mansoni* sirtuins have been expressed throughout the parasitic life cycle and characterized (Lancelot et al., 2013). Initial experiments in adult worms and schistosomula have demonstrated strong effects of hSirt2 inhibitors on both life span and reproduction (Lancelot et al., 2013), and Schiedel et al. found that the smSirt2 IC50 was less than 50 μM (Schiedel et al., 2015). These results suggest that schistosome sirtuins could be potential therapeutic targets and validate screening for selective sirtuin inhibitors as a strategy for the development of new drugs against schistosomiasis.

## Conclusions

In conclusion, epigenetic modifications, including DNA methylation, histone modifications and non-coding RNAs, play important roles in the growth, development and reproduction of schistosomes. DNA methyltransferases, HDACs or miRNAs can be developed as potential drug targets or biomarkers against schistosomiasis. Although great advancements have been achieved in the epigenetic study of schistosomes, additional details remain unknown. In future, a large amount of work must be performed in the epigenetic study of schistosomes. Additionally, epigenetic processes provide potential therapeutic targets for the development of novel therapies against schistosomiasis and other parasitic diseases. These epigenetic modifications are necessary to ensure the development of parasite-selective therapies.

## Author contributions

The author confirms being the sole contributor of this work and approved it for publication.

## Funding

This study was supported by the Agricultural Science and Technology Innovation Program (ASTIP) and Chinese Academy of Agricultural Sciences Central-level Nonprofit Research Institutes Fundamental Research Funds (Grant No. 2016JB05).

### Conflict of interest statement

The author declares that the research was conducted in the absence of any commercial or financial relationships that could be construed as a potential conflict of interest.

## References

[B1] Anderson L. Pierce R. J. Verjovski-Almeida S. (2012). *Schistosoma mansoni* histones: from transcription to chromatin regulation; an *in silico* analysis. Mol. Biochem. Parasitol. 183, 105–114. 10.1016/j.molbiopara.2012.03.001 22414701

[B2] Arrowsmith C. H. Bountra C. Fish P. V. Lee K. Schapira M. (2012). Epigenetic protein families: a new frontier for drug discovery. Nat. Rev. Drug Discov. 11, 384–400. 10.1038/nrd3674 22498752

[B3] Beaver J. E. Waters M. L. (2016). Molecular recognition of Lys and Arg methylation. ACS Chem. Biol. 11, 643–653. 10.1021/acschembio.5b00996 26759915

[B4] Berger S. L. (2002). Histone modifications in transcriptional regulation. Curr. Opin. Genet. Dev. 12, 142–148. 10.1016/S0959-437X(02)00279-4 11893486

[B5] Bergquist R. Utzinger J. Mcmanus D.P. (2008). Trick or Treat: the role of vaccines in integrated schistosomiasis control. PLoS Negl. Trop. Dis. 2:e244. 10.1371/journal.pntd.0000244 18575619 PMC2430529

[B6] Bertin B. Oger F. Cornette J. Caby S. Noël C. Capron M.. (2006). *Schistosoma mansoni* CBP/p300 has a conserved domain structure and interacts functionally with the nuclear receptor SmFtz-F1. Mol. Biochem. Parasitol. 146, 180–191. 10.1016/j.molbiopara.2005.12.006 16427147

[B7] Birney E. Stamatoyannopoulos J. A. Dutta A. Guigó R. Gingeras T. R. Margulies E. H.. (2007). Identification and analysis of functional elements in 1% of the human genome by the ENCODE pilot project. Nature 447, 799–816. 10.1038/nature05874 17571346 PMC2212820

[B8] Bungard D. Fuerth B. J. Zeng P. Y. Faubert B. Maas N. L. Viollet B.. (2010). Signaling kinase AMPK activates stress-promoted transcription via histone H2B phosphorylation. Science 329, 1201–1205. 10.1126/science.1191241 20647423 PMC3922052

[B9] Cai P. Hou N. Piao X. Liu S. Liu H. Yang F.. (2011). Profiles of small non-coding RNAs in *Schistosoma japonicum* during development. PLoS Negl. Trop. Dis. 5:e1256. 10.1371/journal.pntd.0001256 21829742 PMC3149011

[B10] Cai P. Piao X. Hao L. Liu S. Hou N. Wang H.. (2013). A deep analysis of the small non-coding RNA population in *Schistosoma japonicum* eggs. PLoS ONE 8:e64003. 10.1371/journal.pone.0064003 23691136 PMC3653858

[B11] Carneiro V. C. de Abreu da Silva I. C. Torres E. J. Caby S. Lancelot J. Vanderstraete M.. (2014). Epigenetic changes modulate schistosome egg formation and are a novel target for reducing transmission of schistosomiasis. PLoS Pathog. 10:e1004116. 10.1371/journal.ppat.1004116 24809504 PMC4014452

[B12] Carrington J. C. Ambros V. (2003). Role of microRNAs in plant and animal development. Science 301, 336–338. 10.1126/science.1085242 12869753

[B13] Celeste A. Fernandez-Capetillo O. Kruhlak M. J. Pilch D. R. Staudt D. W. Lee A.. (2003). Histone H2AX phosphorylation is dispensable for the initial recognition of DNA breaks. Nat. Cell Biol. 5, 675–679. 10.1038/ncb1004 12792649

[B14] Chen J. Xue Y. (2016). Emerging roles of non-coding RNAs in epigenetic regulation. Sci. China Life Sci. 59, 227–235. 10.1007/s11427-016-5010-0 26825947

[B15] Cheng G. Luo R. Hu C. Cao J. Jin Y. (2013). Deep sequencing-based identification of pathogen-specific microRNAs in the plasma of rabbits infected with *Schistosoma japonicum*. Parasitology 140, 1751–1761. 10.1017/S0031182013000917 23942009

[B16] Cheung W. L. Ajiro K. Samejima K. Kloc M. Cheung P. Mizzen C. A.. (2003). Apoptotic phosphorylation of histone H2B is mediated by mammalian sterile twenty kinase. Cell 113, 507–517. 10.1016/S0092-8674(03)00355-6 12757711

[B17] Deaton A. M. Cook P. C. De Sousa D. Phythian-Adams A. T. Bird A. Macdonald A. S. (2014). A unique DNA methylation signature defines a population of IFN-gamma/IL-4 double-positive T cells during helminth infection. Eur. J. Immunol. 44, 1835–1841. 10.1002/eji.201344098 24578067 PMC4231227

[B18] de Moraes Maciel R. da Costa R. F. de Oliveira F. M. Rumjanek F. D. Fantappié M. R. (2008). Protein acetylation sites mediated by *Schistosoma mansoni* GCN5. Biochem. Biophys. Res. Commun. 370, 53–56. 10.1016/j.bbrc.2008.03.022 18346457

[B19] de Moraes Maciel R. de Silva Dutra D. L. Rumjanek F. D. Juliano L. Juliano M. A. Fantappié M. R. (2004). *Schistosoma mansoni* histone acetyltransferase GCN5: linking histone acetylation to gene activation. Mol. Biochem. Parasitol. 133, 131–135. 10.1016/j.molbiopara.2003.09.005 14668020

[B20] Diao W. Zhou H. Pan W. Liu H. Shen Y. Xu Y.. (2014). Expression and immune characterization of a novel enzyme, protein arginine methyltransferase 1, from *Schistosoma japonicum*. Parasitol. Res. 113, 919–924. 10.1007/s00436-013-3723-6 24343727 PMC3932173

[B21] Dubois F. Caby S. Oger F. Cosseau C. Capron M. Grunau C.. (2009). Histone deacetylase inhibitors induce apoptosis, histone hyperacetylation and up-regulation of gene transcription in *Schistosoma mansoni*. Mol. Biochem. Parasitol. 168, 7–15. 10.1016/j.molbiopara.2009.06.001 19538992

[B22] Fabbri M. Calin G. A. (2010). Epigenetics and miRNAs in human cancer. Adv. Genet. 70, 87–99. 10.1016/b978-0-12-380866-0.60004-6 20920746

[B23] Fendler A. Stephan C. Yousef G. M. Jung K. (2011). MicroRNAs as regulators of signal transduction in urological tumors. Clin. Chem. 57, 954–968. 10.1373/clinchem.2010.157727 21632885

[B24] Flores K. Wolschin F. Corneveaux J. J. Allen A. N. Huentelman M. J. Amdam G. V. (2012). Genome-wide association between DNA methylation and alternative splicing in an invertebrate. BMC Genomics 13:480. 10.1186/1471-2164-13-480 22978521 PMC3526459

[B25] Fneich S. Dheilly N. Adema C. Rognon A. Reichelt M. Bulla J.. (2013). 5-methyl-cytosine and 5-hydroxy-methyl-cytosine in the genome of *Biomphalaria glabrata*, a snail intermediate host of *Schistosoma mansoni*. Parasit. Vectors 6:167. 10.1186/1756-3305-6-167 23742053 PMC3681652

[B26] Fneich S. Théron A. Cosseau C. Rognon A. Aliaga B. Buard J.. (2016). Epigenetic origin of adaptive phenotypic variants in the human blood fluke *Schistosoma mansoni*. Epigenetics Chromatin 9, 27. 10.1186/s13072-016-0076-2 27379173 PMC4931705

[B27] Gerstein M. B. Kundaje A. Hariharan M. Landt S. G. Yan K. K. Cheng C.. (2012). Architecture of the human regulatory network derived from ENCODE data. Nature 489, 91–100. 10.1038/nature11245 22955619 PMC4154057

[B28] Geyer K. K. Chalmers I. W. Mackintosh N. Hirst J. E. Geoghegan R. Badets M.. (2013). Cytosine methylation is a conserved epigenetic feature found throughout the phylum Platyhelminthes. BMC Genomics 14:462. 10.1186/1471-2164-14-462 23837670 PMC3710501

[B29] Geyer K. K. Rodríguez López C. M. Chalmers I. W. Munshi S. E. Truscott M. Heald J.. (2011). Cytosine methylation regulates oviposition in the pathogenic blood fluke *Schistosoma mansoni*. Nat. Commun. 2, 424. 10.1038/ncomms1433 21829186 PMC3265374

[B30] Gregoretti I. V. Lee Y. M. Goodson H. V. (2004). Molecular evolution of the histone deacetylase family: functional implications of phylogenetic analysis. J. Mol. Biol. 338, 17–31. 10.1016/j.jmb.2004.02.006 15050820

[B31] Greiss S. Gartner A. (2009). Sirtuin/Sir2 phylogeny, evolutionary considerations and structural conservation. Mol. Cells 28, 407–415. 10.1007/s10059-009-0169-x 19936627 PMC3710699

[B32] Gutiérrez M. I. Siraj A. K. Khaled H. Koon N. El-Rifai W. Bhatia K. (2004). CpG island methylation in Schistosoma- and non-Schistosoma-associated bladder cancer. Mod. Pathol. 17, 1268–1274. 10.1038/modpathol.3800177 15154012

[B33] Hamidi T. Singh A. K. Chen T. (2015). Genetic alterations of DNA methylation machinery in human diseases. Epigenomics 7, 247–265. 10.2217/epi.14.80 25942534

[B34] Han H. Peng J. Hong Y. Fu Z. Lu K. Li H.. (2015). Comparative characterization of microRNAs in *Schistosoma japonicum* schistosomula from Wistar rats and BALB/c mice. Parasitol. Res. 114, 2639–2647. 10.1007/s00436-015-4468-1 25895062

[B35] Han H. Peng J. Hong Y. Zhang M. Han Y. Liu D.. (2013). MicroRNA expression profile in different tissues of BALB/c mice in the early phase of *Schistosoma japonicum* infection. Mol. Biochem. Parasitol. 188, 1–9. 10.1016/j.molbiopara.2013.02.001 23415751

[B36] Hans F. Dimitrov S. (2001). Histone H3 phosphorylation and cell division. Oncogene 20, 3021–3027. 10.1038/sj.onc.1204326 11420717

[B37] He X. Sai X. Chen C. Zhang Y. Xu X. Zhang D.. (2013). Host serum miR-223 is a potential new biomarker for *Schistosoma japonicum* infection and the response to chemotherapy. Parasit. Vectors 6:272. 10.1186/1756-3305-6-272 24330517 PMC3856452

[B38] Hoy A. M. Lundie R. J. Ivens A. Quintana J. F. Nausch N. Forster T.. (2014). Parasite-derived microRNAs in host serum as novel biomarkers of helminth infection. PLoS Negl. Trop. Dis. 8:e2701. 10.1371/journal.pntd.0002701 24587461 PMC3930507

[B39] Hwang H. W. Mendell J. T. (2006). MicroRNAs in cell proliferation, cell death, and tumorigenesis. Br. J. Cancer 94, 776–780. 10.1038/sj.bjc.6603023 16495913 PMC2361377

[B40] Imhof A. (2006). Epigenetic regulators and histone modification. Brief. Funct. Genomic. Proteomic. 5, 222–227. 10.1093/bfgp/ell030 16951415

[B41] Jabbari K. Bernardi G. (2004). Cytosine methylation and CpG, TpG (CpA) and TpA frequencies. Gene 333, 143–149. 10.1016/j.gene.2004.02.043 15177689

[B42] Jeong K. W. Kim K. Situ A. J. Ulmer T. S. An W. Stallcup M. R. (2011). Recognition of enhancer element-specific histone methylation by TIP60 in transcriptional activation. Nat. Struct. Mol. Biol. 18, 1358–1365. 10.1038/nsmb.2153 22081016 PMC3230772

[B43] Jiang Y. H. Bressler J. Beaudet A. L. (2004). Epigenetics and human disease. Annu. Rev. Genomics Hum. Genet. 5, 479–510. 10.1146/annurev.genom.5.061903.180014 15485357

[B44] Kallestad L. Christensen K. Woods E. Milavetz B. (2014). Transcriptional repression is epigenetically marked by H3K9 methylation during SV40 replication. Clin. Epigenet. 6:21. 10.1186/1868-7083-6-21 25395994 PMC4230732

[B45] Knight M. Ittiprasert W. Arican-Goktas H. D. Bridger J. M. (2016). Epigenetic modulation, stress and plasticity in susceptibility of the snail host, *Biomphalaria glabrata*, to *Schistosoma mansoni* infection. Int. J. Parasitol. 46, 389–394. 10.1016/j.ijpara.2016.03.003 27056272

[B46] Lancelot J. Caby S. Dubois-Abdesselem F. Vanderstraete M. Trolet J. Oliveira G.. (2013). *Schistosoma mansoni* Sirtuins: characterization and potential as chemotherapeutic targets. PLoS Negl. Trop. Dis. 7:e2428. 10.1371/journal.pntd.0002428 24069483 PMC3772001

[B47] Lennartsson A. Ekwall K. (2009). Histone modification patterns and epigenetic codes. Biochim. Biophys. Acta 1790, 863–868. 10.1016/j.bbagen.2008.12.006 19168116

[B48] Lister R. Mukamel E. A. Nery J. R. Urich M. Puddifoot C. A. Johnson N. D.. (2013). Global epigenomic reconfiguration during mammalian brain development. Science 341:1237905. 10.1126/science.1237905 23828890 PMC3785061

[B49] Liu T. Hou L. Zhao Y. Huang Y. (2015). Epigenetic silencing of HDAC1 by miR-449a upregulates Runx2 and promotes osteoblast differentiation. Int. J. Mol. Med. 35, 238–246. 10.3892/ijmm.2014.2004 25405810

[B50] Liyanage V. R. Jarmasz J. S. Murugeshan N. Del Bigio M. R. Rastegar M. Davie J. R. (2014). DNA modifications: function and applications in normal and disease States. Biology (Basel) 3, 670–723. 10.3390/biology3040670 25340699 PMC4280507

[B51] Lo R. Weksberg R. (2014). Biological and biochemical modulation of DNA methylation. Epigenomics 6, 593–602. 10.2217/epi.14.49 25531254

[B52] Lott K. Zhu L. Fisk J. C. Tomasello D. L. Read L. K. (2014). Functional interplay between protein arginine methyltransferases in *Trypanosoma brucei*. Microbiologyopen 3, 595–609. 10.1002/mbo3.191 25044453 PMC4234254

[B53] Mansure J. J. Furtado D. R. de Oliveira F. M. Rumjanek F. D. Franco G. R. Fantappié M. R. (2005). Cloning of a protein arginine methyltransferase PRMT1 homologue from *Schistosoma mansoni*: evidence for roles in nuclear receptor signaling and RNA metabolism. Biochem. Biophys. Res. Commun. 335, 1163–1172. 10.1016/j.bbrc.2005.07.192 16129092

[B54] Marco A. Kozomara A. Hui J. H. Emery A. M. Rollinson D. Griffiths-Jones S.. (2013). Sex-biased expression of microRNAs in *Schistosoma mansoni*. PLoS Negl. Trop. Dis. 7:e2402. 10.1371/journal.pntd.0002402 24069470 PMC3772069

[B55] Marek M. Kannan S. Hauser A. T. Moraes Mourão M. Caby S. Cura V.. (2013). Structural basis for the inhibition of histone deacetylase 8 (HDAC8), a key epigenetic player in the blood fluke *Schistosoma mansoni*. PLoS Pathog. 9:e1003645. 10.1371/journal.ppat.1003645 24086136 PMC3784479

[B56] Marks P. A. Xu W. S. (2009). Histone deacetylase inhibitors: potential in cancer therapy. J. Cell. Biochem. 107, 600–608. 10.1002/jcb.22185 19459166 PMC2766855

[B57] Meng H. Cao Y. Qin J. Song X. Zhang Q. Shi Y.. (2015). DNA methylation, its mediators and genome integrity. Int. J. Biol. Sci. 11, 604–617. 10.7150/ijbs.11218 25892967 PMC4400391

[B58] Mercer T. R. Dinger M. E. Mattick J. S. (2009). Long non-coding RNAs: insights into functions. Nat. Rev. Genet. 10, 155–159. 10.1038/nrg2521 19188922

[B59] Noonan E. J. Place R. F. Pookot D. Basak S. Whitson J. M. Hirata H.. (2009). miR-449a targets HDAC-1 and induces growth arrest in prostate cancer. Oncogene 28, 1714–1724. 10.1038/onc.2009.19 19252524

[B60] Nowak S. J. Corces V. G. (2004). Phosphorylation of histone H3: a balancing act between chromosome condensation and transcriptional activation. Trends Genet. 20, 214–220. 10.1016/j.tig.2004.02.007 15041176

[B61] Oger F. Dubois F. Caby S. Noël C. Cornette J. Bertin B.. (2008). The class I histone deacetylases of the platyhelminth parasite *Schistosoma mansoni*. Biochem. Biophys. Res. Commun. 377, 1079–1084. 10.1016/j.bbrc.2008.10.090 18977200

[B62] Pedersen M. T. Helin K. (2010). Histone demethylases in development and disease. Trends Cell Biol. 20, 662–671. 10.1016/j.tcb.2010.08.011 20863703

[B63] Perrin C. Lepesant J. M. Roger E. Duval D. Fneich S. Thuillier V.. (2013). *Schistosoma mansoni* mucin gene (SmPoMuc) expression: epigenetic control to shape adaptation to a new host. PLoS Pathog. 9:e1003571. 10.1371/journal.ppat.1003571 24009504 PMC3757033

[B64] Peterson C. L. Laniel M. A. (2004). Histones and histone modifications. Curr. Biol. 14, R546–R551. 10.1016/j.cub.2004.07.007 15268870

[B65] Picard M. A. Boissier J. Roquis D. Grunau C. Allienne J. F. Duval D.. (2016). Sex-Biased transcriptome of *Schistosoma mansoni*: host-parasite interaction, genetic determinants and epigenetic regulators are associated with sexual differentiation. PLoS Negl. Trop. Dis. 10:e0004930. 10.1371/journal.pntd.0004930 27677173 PMC5038963

[B66] Portela A. Esteller M. (2010). Epigenetic modifications and human disease. Nat. Biotechnol. 28, 1057–1068. 10.1038/nbt.1685 20944598

[B67] Raddatz G. Guzzardo P. M. Olova N. Fantappié M. R. Rampp M. Schaefer M.. (2013). Dnmt2-dependent methylomes lack defined DNA methylation patterns. Proc. Natl. Acad. Sci. U.S.A. 110, 8627–8631. 10.1073/pnas.1306723110 23641003 PMC3666705

[B68] Ratel D. Ravanat J. L. Berger F. Wion D. (2006). N6-methyladenine: the other methylated base of DNA. Bioessays 28, 309–315. 10.1002/bies.20342 16479578 PMC2754416

[B69] Roquis D. Lepesant J. M. Picard M. A. Freitag M. Parrinello H. Groth M.. (2015). The epigenome of *Schistosoma mansoni* provides insight about how cercariae poise transcription until infection. PLoS Negl. Trop. Dis. 9:e0003853. 10.1371/journal.pntd.0003853 26305466 PMC4549315

[B70] Roquis D. Rognon A. Chaparro C. Boissier J. Arancibia N. Cosseau C.. (2016). Frequency and mitotic heritability of epimutations in *Schistosoma mansoni*. Mol. Ecol. 25, 1741–1758. 10.1111/mec.13555 26826554

[B71] Santosh B. Varshney A. Yadava P. K. (2015). Non-coding RNAs: biological functions and applications. Cell Biochem. Funct. 33, 14–22. 10.1002/cbf.3079 25475931

[B72] Sato F. Tsuchiya S. Meltzer S. J. Shimizu K. (2011). MicroRNAs and epigenetics. FEBS J. 278, 1598–1609. 10.1111/j.1742-4658.2011.08089.x 21395977

[B73] Sayed D. Abdellatif M. (2011). MicroRNAs in development and disease. Physiol. Rev. 91, 827–887. 10.1152/physrev.00006.2010 21742789

[B74] Schiedel M. Marek M. Lancelot J. Karaman B. Almlöf I. Schultz J.. (2015). Fluorescence-based screening assays for the NAD(+)-dependent histone deacetylase smSirt2 from *Schistosoma mansoni*. J. Biomol. Screen. 20, 112–121. 10.1177/1087057114555307 25325257

[B75] Schultz M. D. He Y. Whitaker J. W. Hariharan M. Mukamel E. A. Leung D.. (2015). Human body epigenome maps reveal noncanonical DNA methylation variation. Nature 523, 212–216. 10.1038/nature14465 26030523 PMC4499021

[B76] Sharma A. Singh K. Almasan A. (2012). Histone H2AX phosphorylation: a marker for DNA damage. Methods Mol. Biol. 920, 613–626. 10.1007/978-1-61779-998-3_40 22941631

[B77] Simões M. C. Lee J. Djikeng A. Cerqueira G. C. Zerlotini A. da Silva-Pereira R. A.. (2011). Identification of *Schistosoma mansoni* microRNAs. BMC Genomics 12:47. 10.1186/1471-2164-12-47 21247453 PMC3034697

[B78] Singh R. Pandey P. N. (2015). Molecular docking and molecular dynamics study on SmHDAC1 to identify potential lead compounds against Schistosomiasis. Mol. Biol. Rep. 42, 689–698. 10.1007/s11033-014-3816-z 25663090

[B79] Sombetzki M. Loebermann M. Reisinger E. C. (2015). Vector-mediated microRNA-21 silencing ameliorates granulomatous liver fibrosis in *Schistosoma japonicum* infection. Hepatology 61, 1787–1789. 10.1002/hep.27748 25684616

[B80] Song B. Wang Y. Xi Y. Kudo K. Bruheim S. Botchkina G. I.. (2009). Mechanism of chemoresistance mediated by miR-140 in human osteosarcoma and colon cancer cells. Oncogene 28, 4065–4074. 10.1038/onc.2009.274 19734943 PMC2783211

[B81] Suzuki M. M. Bird A. (2008). DNA methylation landscapes: provocative insights from epigenomics. Nat. Rev. Genet. 9, 465–476. 10.1038/nrg2341 18463664

[B82] Suzuki M. M. Kerr A. R. De Sousa D. Bird A. (2007). CpG methylation is targeted to transcription units in an invertebrate genome. Genome Res. 17, 625–631. 10.1101/gr.6163007 17420183 PMC1855171

[B83] Tang W. W. Dietmann S. Irie N. Leitch H. G. Floros V. I. Bradshaw C. R.. (2015). A unique gene regulatory network resets the human germline epigenome for development. Cell 161, 1453–1467. 10.1016/j.cell.2015.04.053 26046444 PMC4459712

[B84] Tchurikov N. A. (2005). Molecular mechanisms of epigenetics. Biochemistry Mosc. 70, 406–423. 10.1007/s10541-005-0131-2 15892607

[B85] Tsai M. C. Manor O. Wan Y. Mosammaparast N. Wang J. K. Lan F.. (2010). Long noncoding RNA as modular scaffold of histone modification complexes. Science 329, 689–693. 10.1126/science.1192002 20616235 PMC2967777

[B86] Vadaie N. Morris K. V. (2013). Long antisense non-coding RNAs and the epigenetic regulation of gene expression. Biomol. Concepts 4, 411–415. 10.1515/bmc-2013-0014 25436590

[B87] Valeri N. Vannini I. Fanini F. Calore F. Adair B. Fabbri M. (2009). Epigenetics, miRNAs, and human cancer: a new chapter in human gene regulation. Mamm. Genome 20, 573–580. 10.1007/s00335-009-9206-5 19697081

[B88] Wang C. Zhu Y. Chen J. Li X. Peng J. Chen J.. (2014). Crystal structure of arginine methyltransferase 6 from *Trypanosoma brucei*. PLoS ONE 9:e87267. 10.1371/journal.pone.0087267 24498306 PMC3911951

[B89] Wang H. Huang Z. Q. Xia L. Feng Q. Erdjument-Bromage H. Strahl B. D.. (2001). Methylation of histone H4 at arginine 3 facilitating transcriptional activation by nuclear hormone receptor. Science 293, 853–857. 10.1126/science.1060781 11387442

[B90] Wang W. Wang L. Liang Y. S. (2012). Susceptibility or resistance of praziquantel in human schistosomiasis: a review. Parasitol. Res. 111, 1871–1877. 10.1007/s00436-012-3151-z 23052781

[B91] Wen L. Li X. Yan L. Tan Y. Li R. Zhao Y.. (2014). Whole-genome analysis of 5-hydroxymethylcytosine and 5-methylcytosine at base resolution in the human brain. Genome Biol. 15:R49. 10.1186/gb-2014-15-3-r49 24594098 PMC4053808

[B92] Xue X. Sun J. Zhang Q. Wang Z. Huang Y. Pan W. (2008). Identification and characterization of novel microRNAs from *Schistosoma japonicum*. PLoS ONE 3:e4034. 10.1371/journal.pone.0004034 19107204 PMC2603315

[B93] Zhong X. Isharwal S. Naples J. M. Shiff C. Veltri R. W. Shao C.. (2013). Hypermethylation of genes detected in urine from Ghanaian adults with bladder pathology associated with *Schistosoma haematobium* infection. PLoS ONE 8:e59089. 10.1371/journal.pone.0059089 23527093 PMC3601097

[B94] Zhu L. Dao J. Du X. Li H. Lu K. Liu J.. (2015). Altered levels of circulating miRNAs are associated *Schistosoma japonicum* infection in mice. Parasit. Vectors 8, 196. 10.1186/s13071-015-0806-5 25885182 PMC4391475

[B95] Zhu L. Zhao J. Wang J. Hu C. Peng J. Luo R.. (2016). MicroRNAs Are Involved in the regulation of ovary development in the pathogenic blood fluke *Schistosoma japonicum*. PLoS Pathog. 12:e1005423. 10.1371/journal.ppat.1005423 26871705 PMC4752461

